# 2525. Identification of lead compounds, belonging to 26-membered thiopeptide antibiotics for new anti-tuberculosis therapeutic agents

**DOI:** 10.1093/ofid/ofad500.2143

**Published:** 2023-11-27

**Authors:** Young-Jin Son, Hee-Jong Hwang, Clovis Shyaka, Dahyun Kim, Jusuk Lee, Seokyong Eum, Sun-Kyung Lee

**Affiliations:** A&J Science, daegu, Taegu-jikhalsi, Republic of Korea; A&J Science, daegu, Taegu-jikhalsi, Republic of Korea; A&J Science, daegu, Taegu-jikhalsi, Republic of Korea; A&J Science, daegu, Taegu-jikhalsi, Republic of Korea; A&J Science, daegu, Taegu-jikhalsi, Republic of Korea; International Tuberculosis Research Center, Changwon, Kyongsang-namdo, Republic of Korea; International Tuberculosis Research Center, Changwon, Kyongsang-namdo, Republic of Korea

## Abstract

**Background:**

Tuberculosis (TB) is caused by a bacterium called *Mycobacterium tuberculosis (Mtb)*. The incidence of TB patients is increasing globally and the wide spread of multi- and extensively drug-resistant TB poses a significant burden to patients. This situation calls for an urgent medical need to develop new anti-TB drugs. Through our proprietary medicinal chemistry platform on D-series 26-membered thiopeptide, we have identified a few lead compounds, such as AJ-099 and AJ-206 that exert potent activity against multi-drug resistant TB strains.

**Methods:**

*In vitro* minimal inhibitory concentration (MIC) was measured and a Mtb-infected human macrophage model was used to evaluate anti-mycobacterial activity of our compounds in drug-sensitive or multidrug-resistant TB isolates. Cellular toxicity and some *in vitro* ADME tests were also performed and examined.

**Results:**

We found that our lead compounds exert potent anti-TB activity on H37Rv(MIC: 0.125-0.5 μg/mL) and showed similar MIC levels in multidrug-resistant clinical isolates. In the macrophage infection model, AJ-099 and AJ-206 showed comparable antimycobacterial effects to isoniazid. These compounds showed no cytotoxicity, relatively safe ADME properties, and no hERG inhibition.

In vitro antibacterial activity in multi-drug resistant M. tuberculosis strains

**Figure:**
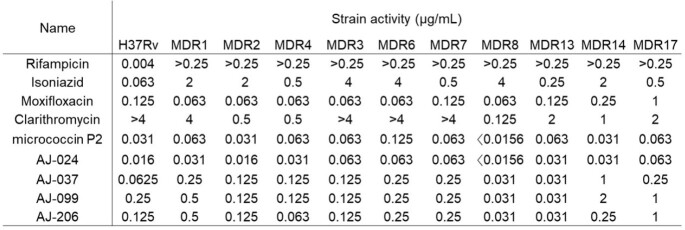

Antibacterial activity in macrophage infection model

**Figure:**
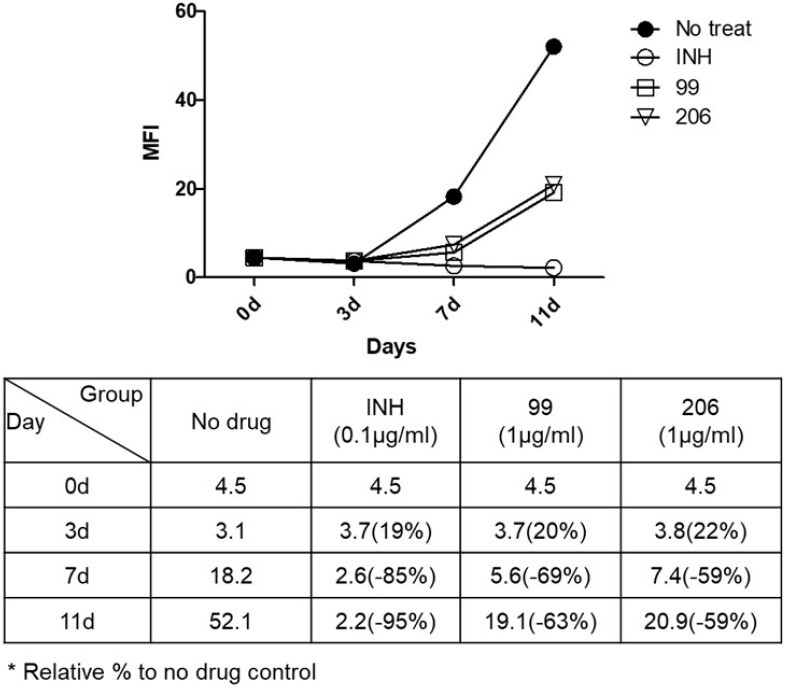

In vitro ADMET properties of lead compounds

**Figure:**
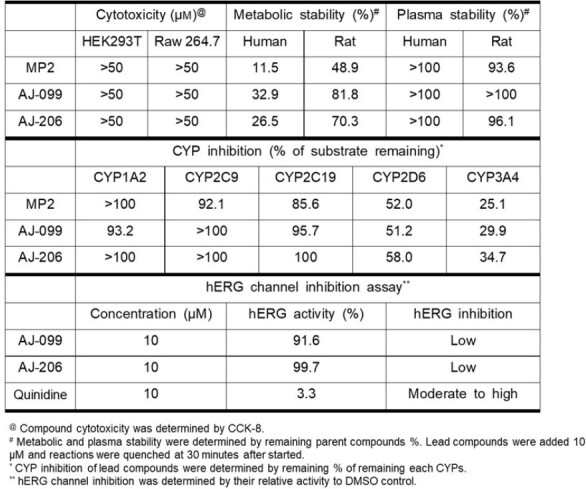

**Conclusion:**

AJ-099 and AJ-206 may be potential anti-TB therapeutic agents that possess novel modes of action, low cardiac and cellular toxicities.

**Disclosures:**

**Young-Jin Son**, A&J Science: Employee of A&J Science|A&J Science: Ownership Interest|KHIDI: Grant/Research Support **Hee-Jong Hwang, PhD**, A&J Science: Stocks/Bonds|KHIDI: Grant/Research Support **Clovis Shyaka, n/a**, A&J Science: Employee of A&J Science **Dahyun Kim, n/a**, A&J Science: Employee of A&J Science **Jusuk Lee, Ph.D.**, A&J Science: Employee of A&J Science

